# What Are Kinematic and Kinetic Differences between Short and Parallel Turn in Alpine Skiing?

**DOI:** 10.3390/ijerph18063029

**Published:** 2021-03-16

**Authors:** Ivan Bon, Mateja Očić, Vjekoslav Cigrovski, Tomislav Rupčić, Damir Knjaz

**Affiliations:** Laboratory for Sports Games, Faculty of Kinesiology, University of Zagreb, 10000 Zagreb, Croatia; mateja.ocic@kif.unizg.hr (M.O.); vjekoslav.cigrovski@kif.unizg.hr (V.C.); tomislav.rupcic@kif.unizg.hr (T.R.); damir.knjaz@kif.unizg.hr (D.K.)

**Keywords:** recreational skiing, biomechanics, pressure insoles, Xsens motion capture system, sport performance analysis

## Abstract

There are numerous programs worldwide adapted for alpine ski beginners and they all share the same primary goal—inclusion of skiing beginners in alpine ski schools. The final elements of ski school taught in the parallel skiing technique are parallel turn and short turn. Synchronized analysis of kinetic and kinematic parameters of the parallel turn (PT) and short turn (ST) was conducted to determine the main biomechanical differences from a standpoint of foot pressure and lower limb angles. Both elements were performed by nine male ski instructors (age 33.4 ± 8.62, height 179.52 ± 5.98 cm, weight 78.6 ± 8.88 kg). Kinetic and kinematic analysis was conducted on 180 turns, 90 for each element. Differences in kinetic and kinematic parameters between parallel and short turns were tested by a paired *t*-test. The main findings of our study are determined differences in the ratio of pressure distribution on the inside and the outside foot and differences in kinematic parameters of the outside leg between elements. The mentioned analysis can provide an objective insight into the complexity of each element and provide guidelines for teaching process of those elements. This study determined the reasons for higher complexity of ST compared to PT based on the objective evaluation of biomechanical factors.

## 1. Introduction

Alpine skiing is one of the most popular winter recreational activities [1]. The aim for each recreational skier is to enjoy skiing and be safe, which can be achieved by participating in efficient ski schools [2,3]. There are numerous programs worldwide that have been adapted for alpine ski beginners, and, although different, they all share the same primary goal—inclusion of skiing beginners in alpine ski schools [4,5].

Each ski school program is based on general principles of learning certain motor skills. Considering that skiing is a specific activity which consists of unnatural and uncommon movements for the human body, the principle of gradual learning is crucial. Ski elements are taught starting with simple ones, executed under slow speeds, and then progressing gradually towards more complex elements, which are executed under higher speeds [6,7]. Once skiers learn the basic methods of controlling speed, they learn how to turn by using the changes in the center of mass and ground reaction force (GRF) while maintaining a suitable posture in order to control the direction [8].

The main unit which is continuously executed while skiing is the ski turn. According to authors who studied alpine skiing, it can be divided in two or three distinct phases [8,9,10]. Starting from the first phase, a skier must perform specific movements in a timely manner, adopt the optimal body position for the ski slope and also impose pressure on the outside ski. This will result with a ski turn whose duration, speed and direction are determined by the skier itself. A ski turn is a very complex action which leads to regular changes in the direction of the turn to the right and to the left side. The frequency of the turns, their total duration, the radius of the turn and the speed of movement in relation to external factors (for example the quality of the snow surface, inclination of the slope, etc.) are all variables which considerably influence the outcome of the actual turn [11,12]. Although the key principles are identical, the recreational level ski turn differs from the turns executed by a ski racer. The main differences between the two are the speed, tempo and rhythm. In recreational skiing, the skier determines all of the above together with the line of descent. In ski racing, on the other hand, these elements are determined by the gates set up on the slope. 

The final elements of ski school taught in the parallel skiing technique are the parallel turn (PT) and short turn (ST). These elements are also used the most by recreational skiers while conquering the ski slope. Furthermore, their biomechanical characteristics are similar [13,14]. While describing ST, ski instructors often use the phrase “chopped unfinished PT”. PT is executed in a wide corridor and the duration of the turn is longer, which enables a medium level recreational skier to control the speed and direction more easily. It is usually performed on moderately steep and wide slopes. ST is executed in a narrow corridor and duration of the turn is shorter, leaving the skier with no time to think about the turn performance. It requires automatization of movements and it is performed on the steep slopes, usually on crowded slopes in order to avoid collisions. Therefore, based upon ski instructors’ experiences, it is taught after PT. The main difference between these types of turns is the rhythm at which they are executed, and the speed also varies [13].

Although numerous descriptions of the alpine skiing technique have been published, relatively little is yet known about the biomechanical factors that influence competitive performance [15]. The number of studies focused on recreational level alpine skiing are even more limited, especially regarding the biomechanical characteristics of ski elements.

Kinematic and kinetic analysis can provide a clear insight in the complexity of each movement while performing a ski turn, and it also allows comparisons between different types of turns [14,16,17]. Biomechanical analysis of winter sports is very challenging due to environmental conditions, which are hard to simulate in laboratory conditions [18]. Therefore, it is important to use equipment that provides accurate data capture during on-field testing. A Xsens motion capture system is used for determining kinematic parameters while performing simple or complex movements and for assessing full body motion [10,18,19,20,21]. Investigators have recently used pressure insoles (Novel, Pedar) during alpine skiing to better understand the relationship between kinematic and kinetic parameters, and their effects on performance, training and mechanisms of injury [22,23,24,25,26,27]. Pressure insoles can also be used outside of the laboratory and can be a powerful tool for precisely assessing the load distribution on feet during the performance of various types of ski turns [28]. 

Ski schools and ski coaches give their students and athletes feedback about their skiing performance, mainly based on subjective assessments. The Xsens system and pressure insoles can precisely determine biomechanical factors that influence the performance of each ski turn. They could therefore provide a better understanding of ski elements and consequently make the ski school teaching process and the development of young skiers more efficient. 

For that reason, synchronized analysis of kinetic and kinematic parameters of ST and PT was conducted to determine the main biomechanical differences from a standpoint of foot pressure and lower limbs angles. This analysis can provide an objective insight into the complexity of each element and offer some guidelines for the teaching process. Also, the results can help to clarify whether the usual methodical order of teaching the ST after PT is justified. 

We hypothesized that the ST is more complex in terms of the technique used, which is manifested in higher foot pressure and a different ratio of pressure distribution regarding the outside and inside foot. Also, regardless of their similarities in specific movements, we assumed there would be differences in kinematic parameters of lower limbs during the performance of ST and PT. 

## 2. Materials and Methods

Participants: The sample consisted of nine male ski instructors (age 33.4 ± 8.62, height 179.52 ± 5.98 cm, weight 78.6 ± 8.88 kg). Participants did not have any prior injuries that could affect skiing techniques or kinetic and kinematic variables in both ski elements. All participants were provided with a detailed explanation of the study procedures and they gave their written informed consent prior to the measuring procedure. The Faculty of Kinesiology, University of Zagreb (Croatia) Ethics Committee approved the study, which was performed following the ethical standards of the Declaration of Helsinki. 

Variables: Analysis of kinetic and kinematic parameters was conducted on two elements of parallel alpine ski school (PT and ST). A total of 180 turns were analyzed (90 in each element). The variable sample included 10 variables in total. The following kinetic variables were measured: maximum force of the right foot in the left turn (Max_F_R_LT); maximum force of the left foot in the left turn (Max_F_L_LT); maximum force of the left foot in the right turn (Max_F_L_RT); maximum force of the right foot in the right turn (Max_F_R_RT). All results are shown in newtons (N). The following kinematic variables were obtained (180° = knee and hip fully extended): angle of the right hip flexion in the left turn (Hip_R_LT); angle of the right knee flexion in the left turn (Knee_R_LT); angle of the left knee flexion in the left turn (Knee_L_LT); angle of the left hip flexion in the right turn (Hip_L_RT); angle of the left knee flexion in the right turn (Knee_L_RT); angle of the right knee flexion in the right turn (Knee_R_RT). All results are shown in degrees (°). 

**Figure 1:**
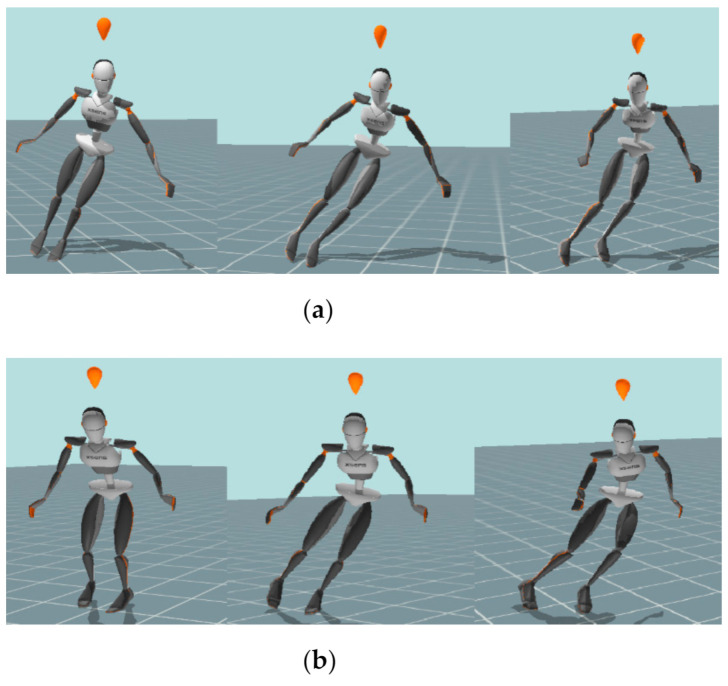
(**a**) Kinogram of a parallel turn (PT) from the first to the third phase of the turn. (**b**) Kinogram of a short turn (ST) from the first to the thirdphase of the turn.

All data were obtained in the final phase of each turn in both elements, precisely in the moment when skiers’ center of mass was at the lowest point and just before releasing the pressure on the outside ski (Figure 1a,b).

To measure the kinetic variables, insoles designed for pressure detection (Novel, Pedar) were used. Insoles are thin and light (2 mm), so they have a minimal influence on a skier’s performance during testing, which is especially important during more dynamic skiing, i.e., performance of advanced elements of ski techniques. For the purposes of this study, the sampling rate was set at 100 Hz. Data were derived from the corresponding Novel software (Loadsol analysis 25.3.6) A previous study has confirmed the reliability and validity of Novel pressure insoles for analyzing foot pressure in alpine skiing [26,27]. 

Kinematic parameters were measured by a Xsens MVN Link inertial suit system. The system consists of 17 three-dimensional accelerometers/gyroscopes/magnetometers, battery and it was set at a 240 Hz output rate. Data were derived from the corresponding MVN BIOMECH software (Xsens, MVN Studio 4.4, firmware version 4.3.1, Enschede, The Netherlands). Previous studies have confirmed the reliability and validity of the Xsens kinematic suit for analyzing kinematic parameters (joint angles) in alpine skiing [19,20,21].

Protocol of investigation: All instructors underwent the same protocol: measuring of anthropometric characteristics, adjusting the Xsens suit, adjusting pressure insoles inside the ski boots, a free skiing warm up run, a trial run in corridor for PT, measuring the performance of PT, a trial run in a corridor for ST and measuring the performance of ST.

Anthropometric characteristics were used for calibration of the Xsens system. It was used according to the standard of procedure advised by the manufacturer (Xsens technologies B.V., Netherlands). Standard calibration of pressure insoles was performed according to manufacturers’ advice (Novel GmBh, Munich). In order to synchronize both kinetic and kinematic systems, all turns were recorded with a video camera (Panasonic GH 5). For the purpose of synchronization, participants were asked to alternately lift their skis two times off the snow surface just before starting their descent. That was the starting moment when the time lapse of both systems was aligned.

**Figure 2:**
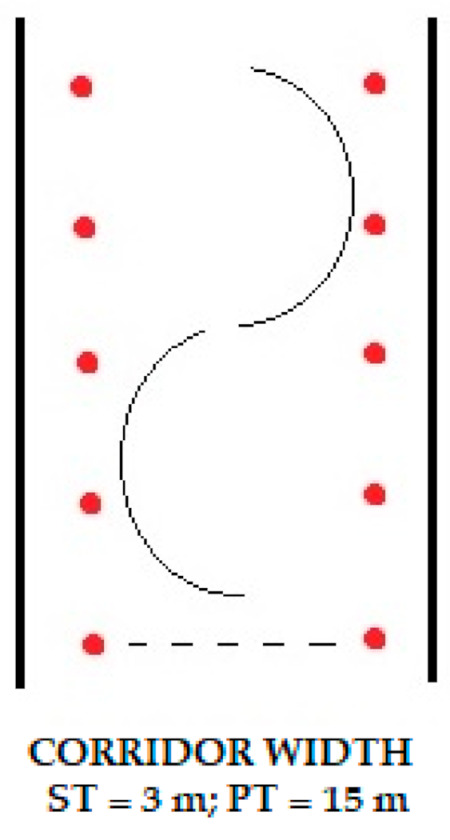
Display of the corridor.

Overall testing was conducted for two days (four instructors on the first day and five on the following day). The study was performed in the morning hours to secure better snow conditions. The average incline of the slope was 20°. Participants had identical skiing equipment (slalom skis and ski boots) during demonstration of both elements of ski technique. Both ski elements were performed on the selected part of the terrain, taking into consideration the characteristics of specific elements, in previously defined corridor of 15 m wide in the case of PT and 3 m wide in the case of ST (Figure 2). Participants received instructions to perform standard PT and ST in the same way to that demonstrated in alpine ski school. Each turn had to be performed from the left to the right end of the corridor, and vice versa. Each ski instructor performed 12 turns in each observed element. Due to some mistakes in motor movement (skidding, sliding, etc.) several turns were excluded from further analysis.

Statistical analysis: For the statistical analysis, program Statistica version 13.5.0.17 (manufacturer: TIBCO Software Inc, Palo Alto, CA, USA; release date: November 2018) was used. Basic descriptive parameters were calculated for all measured variables. The normality of the data distribution was tested by the Kolmogorov–Smirnov test. For the detection of differences between the elements, a paired *t*-test was used. The results were considered significant when *p* < 0.05. With the use of the G*power program, the sample size (number of turns) was calculated (*n* = 90) that was needed for a measurement procedure with statistical significance of *p* ˂ 0.05; statistical power 0.8; effect size 0.3 and 2 groups.

## 3. Results

Basic descriptive parameters of each tested variable along with results of paired *t*-test are presented in Table 1.

Table 1 shows that significant differences between two observed ski elements can be determined through six measured variables. Four of the mentioned variables are related to foot pressure on snow surface. In the left turn, there was a significant difference in maximum pressure force of the outside and the inside foot between ST and PT (*p* = 0.000; *p* = 0.000). Pressure force values of the outside foot in ST are higher than in PT (Max_F_R_LT = 1141.82 N; Max_F_R_LT = 834.63 N). The force on the outside foot of ST represents 1.45 of mean body weight (MBW) of tested participants while the force of PT is 1.06 MBW. The force of the inside foot in the left turn was also higher while performing ST compared to PT (Max_F_L_LT = 707.68 N; Max_F_L_LT = 257.96 N). In relation to body weight, the measured force in ST was 0.92 MBW was 0.33 MBW in PT. 

**Figure 3:**
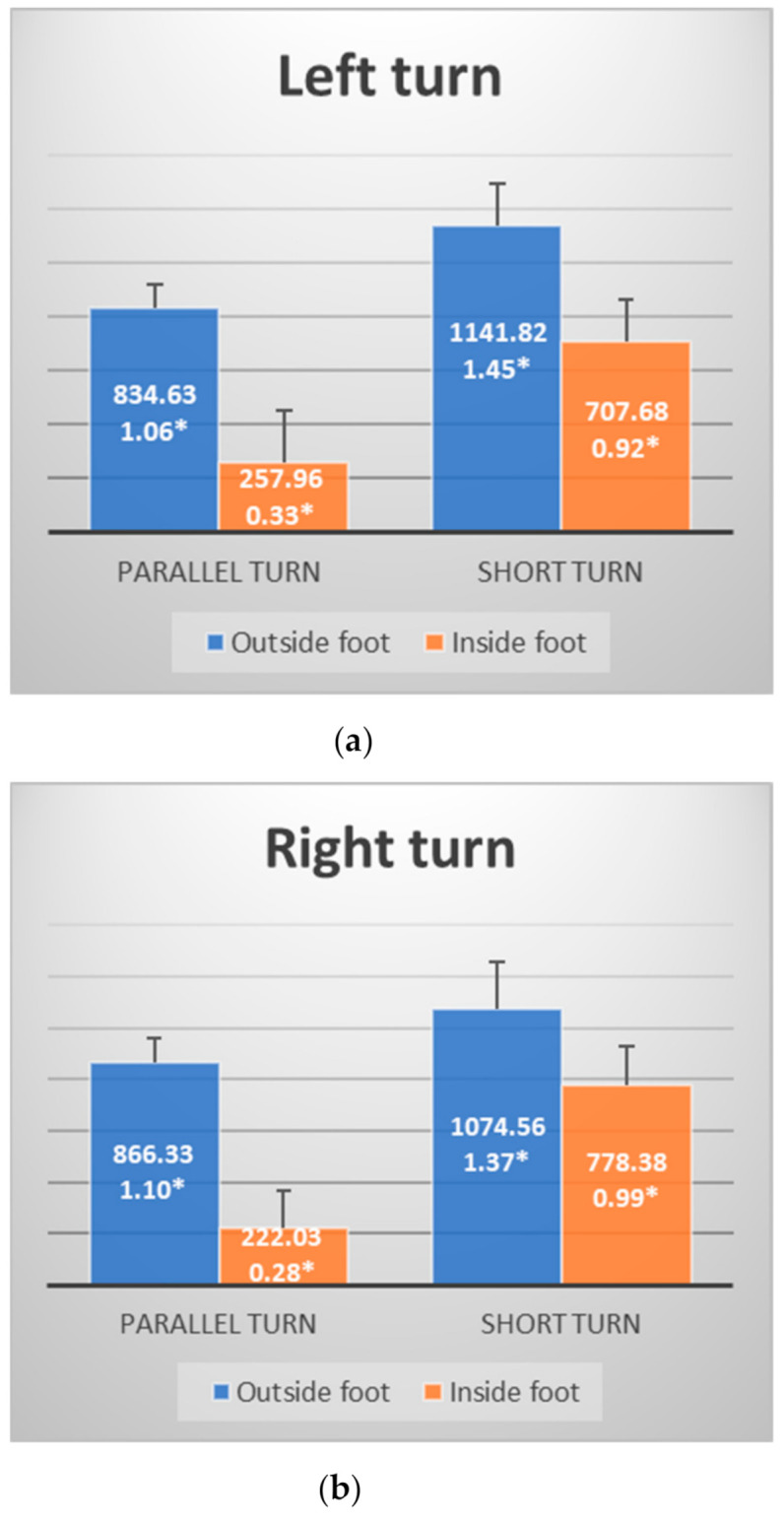
(**a**) pressure forces in the left turn expressed in Newtons and mean body weight (marked with *); (**b**) pressure forces in the right turn expressed in Newtons and mean body weight (marked with *).

In the right turn, a significant difference between the two observed elements was also determined in maximal force pressure of the outside and the inside foot (*p* = 0.000, *p* = 0.000). The pressure force values of the outside foot in ST are higher than in PT (Max_F_L_RT =1074.56 N; Max_F_L_RT = 866.33 N). In relation to body weight, the measured force in ST was 1.37 MBW and was 1.10 MBW in PT. Furthermore, in ST the measured force of the inside foot was higher than in PT (Max_F_R_RT = 778.38 N; Max_F_R_RT = 222.03 N). In relation to body weight, the measured force in ST was 0.99 MBW and was 0.28 MBW in PT. Figure 3 presents pressure forces in both elements in relation to MBW (Figure 3a,b).

Significant differences regarding kinematic parameters between the two observed elements were found in the right knee flexion angle (*p* = 0.002) in the left turn and in the left knee flexion angle (*p* = 0.036) in the right turn. At the end of each turn, the knee was more flexed i.e., the skier was in a lower position while performing the ST element (PT-Knee_R_LT = 141.46°, Knee_L_RT = 148.74°; ST-Knee_R_LT = 138.41°, Knee_L_RT = 146.12°). 

## 4. Discussion

The aim of this study was to determine the differences in foot pressure and lower limbs angles between the two most commonly used elements in the parallel alpine skiing school. The main findings of our study are differences in the ratio of the inside and the outside foot pressure distribution and differences in kinematic parameters of the outside leg between the elements. Those findings are in accordance with previously stated hypotheses. ST, based on the results, is a more complex element and its acquisition should therefore follow the PT.

There is a statistically significant difference between ST and PT in foot pressure forces, both on the outside and the inside foot (*p* = 0.000). Mean values of maximum pressure were higher in ST than in PT, in both feet, while performing elements on both the right and the left side. The ratio of foot pressure between the inside and the outside foot differed in the two observed elements. Foot pressure, although mainly on the outside foot, was significantly higher on the inside foot in ST when comparing the two elements (ST-Max_F_L_LT = 707.68 N, Max_F_R_RT = 778.38 N; PT-Max_F_L_LT = 257.96 N, Max_F_R_RT = 222.03 N). Higher foot pressure forces of the inside foot in ST could be explained by a higher complexity of the ski element. ST is executed in a more “choppy” fashion and, as was mentioned previously, it is commonly referred to as a short unfinished PT. Therefore, each phase of the turn and consequently the whole turn is performed in a shorter time interval. Each specific movement is executed faster and is performed automatically without preparing for each phase. Body weight is transferred faster from one to another outside ski. During that movement, a skier uses GRF in order to facilitate and speed up the transfer of the pressure from the outside leg to the new outside leg in the following turn. To ease the transfer movement and make it more efficient, the skier must use the inside foot, which will help him to master generated forces. Furthermore, at the end of the turn, the skier cannot control the speed by executing an uphill turn since the turn is unfinished by definition. High values of GRF detected at the end of the ST in this research are just another factor influencing the complexity of speed control. The steepness of the slopes increases the danger of injuries caused by a fall. Collision among skiers is dangerous, especially on steeper slopes where it is harder to stop the sliding after a fall. Therefore, ST is usually executed on the steep slopes by experienced skiers who mastered the speed control at the end of the turn. By using a narrow corridor, a skier minimizes the risk of collision with other skiers. On the other hand, when performing PT, the duration of the turn is longer and there is more time for adjusting movements in each phase of the turn. Also, a skier has more time to apply pressure on the ski surface at the right moment [29]. Generated forces measured in our research were also lower on average i.e., they were easier to master, and use to control the speed and direction of the skis. The speed can also be controlled when finishing the turn by performing an uphill turn. Consequently, PT is more suitable for skiers with an intermediate level of skiing techniques in a wider corridor and on moderately steep slopes. It enables them to perform the turn in its full form.

Kim and Kim [8] compared ground reaction forces between ST and PT performed by ski instructors. They reported a significantly greater increase in GRF during the third phase of the turn while performing ST (*p* < 0.05), which is in accordance with data obtained in our research. The authors concluded the main reason for this difference was the lesser time in which the ST must be completed, without skidding which is manifested by an increase of GRF. The increase of GRF is also achieved by a higher edging angle, especially in the last phase of the turn. They suggested that while acquiring ST, the last phase of the turn should be short with a fast transfer of body mass to the other outside ski. While performing PT, the skier learns how to control the center of mass, pressure distribution and edging angle. After mastering the above mentioned techniques, the skier can achieve a high edging angle and utilize a high GRF to transfer the pressure to the new outside foot.

Force distribution analysis was conducted on elite ski instructors while performing slalom (SL) and giant slalom (GS) turn in a study of Lamontagne [25]. His findings were similar regarding total maximum force, which was higher for the outside foot than the inside foot independently of the type of the turn. However, average forces for both turns were higher than in our research. In his study, the average force on the outside foot represented two times BW while the force on the inside is 1.2 BW. In our study, the force on the outside foot was 1.26 BW and on the inside was 0.63 BW. As already stated, race turns executed with gates are an upgrade of basic ski school elements PT and ST. Although similar, the main goals of recreational level skiing and ski racing differ greatly one from another. A higher speed, tempo and rhythm of the turn are characteristic of ski racing. Consequently, higher forces are expected when comparing SL to ST and GS to PT. This was also confirmed in research conducted by Kroll et al. [30]. PT compared to GS is executed in a slower speed and both the GRF and foot pressure are lower, so the risk of injuries is minimized. Also, pressure distribution on the inside and the outside leg is similar, the duration of each phase and the whole turn are similar and also the width of the corridor in which the turn is generally performed is similar. Therefore, PT can be a very useful tool and serve as a methodical exercise during training of GS in preseason. The same can be stated for ST and SL. It is common for ski instructors to work with ski racers in preseason when the athletes are perfecting basic positions and want to “feel” an optimal pressure distribution.

In a research conducted by Falda–Buscaiot et al. [23], the ratio of pressure distribution is in accordance with our results when observing PT even though they conducted research on GS. It represents a 75%/25% distribution on the outside and the inside foot and in our study, it is 78%/22% on average. In the mentioned research, the participants were youth ski racers. It is common for the youngest athletes to train and compete only in GS. Only after mastering GS do they start to train SL. This is in accordance with the teaching process of recreational level skiing. After perfecting PT, recreational level skier can start to learn ST. The same process is followed by young ski athletes. After mastering ski elements from most basic ones to PT and finally ST, they can start learning GS and afterwards SL. When perfecting the basic position and pressure distribution in GS, it is necessary for professional alpine ski racers and young athletes to start with PT. The same applies for SL and ST. Based on the mentioned studies and data presented in our research regarding pressure distribution, it can be concluded that ST is more complex and should be taught only after mastering PT.

Kinematic analysis showed significant differences between turns in knee angle values of the outside leg (*p* = 0.002, *p* = 0.036). In ST it is very important to utilize the ground reaction force at the end of the turn in order to be faster in transition. Consequently, the skier must be in a lower position, i.e., his knee angle of the outside leg is smaller (Knee_R_LT = 138.41°, Knee_L_RT = 146.12°). Kröll et al. [31] investigated quadriceps muscle activity in alpine skiing and knee angle values during the turn. They found that smaller knee angle values enable the skier to generate a higher force and be more efficient when transferring pressure to the new outside leg. This research is in accordance with the obtained kinematic results in our research and confirms the conclusions of the conducted kinetic analysis. Balanced position with the proper flexion of joints and aligned lower body segments are a precondition for optimal pressure distribution. Since the corridor in which ST is performed is narrow, the upper body should be as still as possible while all the movements are performed in the lower body. Therefore, the knee angle is expected to be lower during the third phase of the turn in order to ease the transfer of pressure to the new outside leg in the next turn.

In the previously cited study conducted by Kroll et al. [30], during GS and SL skiing, there were similarities with the results obtained in our research regarding knee angle values of the outside leg. Authors found a smaller knee angle while performing SL, meaning that during GS, the outside leg is more extended. Observed differences between knee angles of the outside leg in SL and GS were explained from a functional perspective by a greater whole-body inclination in GS. It is possible to make similar conclusion while observing ST and PT. Regarding complexity, it is more demanding for a skier to separate the movements of the upper and lower body, which is more the case while performing ST than PT. This outcome is more related to performing ST and SL due to a narrow corridor and the “unfinished” last phase of the turn. Therefore, both kinematic and kinetic data indicate that the turns performed in a narrow corridor (ST and SL) are more complex than the ones performed in a wide corridor (PT and GS).

Most of the kinetics and kinematics research addressed the issues of force application and changes in joint angles during dynamic performances of racers [10,15,23,25,29,30,32]. The findings of those studies focusing on racers can be applied to recreational level skiers, although, as stated above, differences between the two types of skiing exist and one should be cautious with conclusions. The number of articles analyzing kinetic and kinematic parameters in recreational level skiing is limited, although the number of recreational level skiers is increasing worldwide. Therefore, the demand for such studies that would include both kinetic and kinematic parameters of alpine ski school elements is growing.

Information about the timing of specific body movements which ensure optimal control of direction and speed could be crucial for accomplishing the aim of each alpine ski school—to enable skiers to be independent in mastering complex skiing elements. This research determined the reasons for higher complexity of ST compared to PT based on the objective evaluation of biomechanical factors that influence the performance of each element. Therefore, we confirmed that current methodical order of teaching ST after PT is justified. Based on our research, the turns performed in a narrow corridor are more complex due to their pressure distribution and lower body joint flexion, which is probably caused by a greater separation of movement in upper and lower body. Ski instructors and coaches should follow the methodical process from the beginning of teaching. All technical elements should be demonstrated and taught in a wide corridor with longer duration of each turn phase. This way, the students and athletes will have the necessary time to process and perform all the movements. Future studies should be focused on observing biomechanical factors of all the elements performed in narrow and wide corridors. Only then would it be possible to make a conclusion about the reasons beneath the complexity of performing a turn in a narrow corridor.

### Limitations

The presented study is focused on kinematic and kinetic analysis of only two elements of alpine ski school. To make a conclusion about the complexity of elements and teaching methodology, it would be necessary to conduct measurements of each element included in alpine ski school program and every methodical exercise for teaching those elements. This would give ski instructors and coaches much more assurance when deciding upon the choice and the order of methodical exercises to use in the most efficient way to upgrade the teaching process of ski elements. Furthermore, it would be useful to determine pressure distribution on different areas of foot during performing different phases of the turn.

## 5. Conclusions

The results of the conducted synchronized analysis of ski elements determined different ratio of pressure distribution between the elements. The pressure on the inside leg is relatively higher in ST compared to PT. Kinematic analysis pointed out differences in the knee angle of the outside leg, which has a role of controlling the speed and direction during descent. ST is more complex and skiers can only safely perform ST after mastering the optimal and balanced position with pressure dominantly on the outside ski recreational level alpine.

Similar findings were determined in various alpine skiing studies focused on kinetic and kinematic analysis. Differences between those studies and the present one are participants and the type of analyzed turns. Although there are similarities between SL and GS and some elements of alpine ski school, it is important to be cautious when making conclusions about recreational skiing based on ski racers and their turns. 

Studies focused on alpine ski schools are necessary to determine crucial biomechanical factors influencing the performance of each element. It is important that ski instructors have that information because it can serve as a guideline for adapting their teaching process and make it more efficient, which will consequently reduce injuries.

## Figures and Tables

**Table 1:** Basic descriptive statistical parameters and *t*-test for variables PT and ST.

Variable	Parallel Turn Mean ± SD	Short Turn Mean ± SD	*p*
Max_F_R_LT (N)	834.63 ± 83.52	1141.82 ± 151.70	0.000 *
Max_F_L_LT (N)	257.96 ± 193.82	707.68 ± 155.21	0.000 *
Hip_R_LT (°)	164.99 ± 4.71	163.68 ± 5.76	0.240
Knee_R_LT (°)	141.46 ± 3.70	138.41 ± 5.25	0.002 *
Knee_L_LT (°)	115.69 ± 6.23	113.24 ± 9.39	0.148
Max_F_L_RT (N)	866.33 ± 91.29	1074.56 ± 183.70	0.000 *
Max_F_R_RT (N)	222.03 ± 143.64	778.38 ± 146.92	0.000 *
Hip_L_RT (°)	166.20 ± 2.53	165.58 ± 2.66	0.264
Knee_L_RT (°)	148.74 ± 3.46	146.12 ± 7.51	0.036 *
Knee_R_RT (°)	117.49 ± 5.50	115.55 ± 7.02	0.149

* *p* < 0.05; Max_F_R_LT—maximum force of the right foot in a left turn; Max_F_L_LT—maximum force of a left foot in the left turn; Hip_R_LT—angle of the right hip flexion in a left turn; Knee_R_LT—angle of the right knee flexion in a left turn; Knee_L_LT—angle of the left knee flexion in a left turn; Max_F_L_RT—maximum force of the left foot in a right turn; Max_F_R_RT—maximum force of the right foot in a right turn; Hip_L_RT—angle of the left hip flexion in a right turn; Knee_L_RT—angle of the left knee flexion in a right turn; Knee_R_RT—angle of the right knee flexion in a right turn; (N)—newton; (°)—degrees.

## Data Availability

The data presented in this study are available on request from the corresponding author. The data are not publicly available due to its huge size and participants’ privacy protection.
